# Pollination in the Rainforest: Scarce Visitors and Low Effective Pollinators Limit the Fruiting Success of Tropical Orchids

**DOI:** 10.3390/insects12100856

**Published:** 2021-09-23

**Authors:** Hortensia Cabrera Reyes, David Draper, Isabel Marques

**Affiliations:** 1 Universidad Yachay Tech, Hacienda San José s/n, 100115 San Miguel de Urcuquí, Ecuador; 2 Centre for Ecology, Evolution and Environmental Change, National Museum of Natural History and Science, Universidade de Lisboa, 1250-102 Lisboa, Portugal; ddmunt@gmail.com; 3 Forest Research Centre (CEF), Plant-Environment Interactions and Biodiversity Lab (PlantStress & Biodiversity), Instituto Superior de Agronomia (ISA), Universidade de Lisboa, Tapada da Ajuda, 1349-017 Lisbon, Portugal

**Keywords:** deception, orchids, pollination, rainforest, reproduction, tropical pollinators

## Abstract

**Simple Summary:**

The ability to distinguish between mere flower visitors and effective pollinators is of crucial importance to understand pollination ecology. Inefficient and scarce pollinators, coupled with unsuitable abiotic conditions, might explain a failure in plant reproductive success. Here, we quantified the levels of fruit set and tested the dependency on pollinators in 10 orchid species occurring in the tropical rainforest. We show that all species were pollinator-dependent with pollination limitation occurring in all the 20 studied populations. In fact, in 87% of our observation period no visitor was seen on the flowers studied. Floral visitors included flies, butterflies, bees, and hummingbirds although only a small number were performing effective pollinations. We conclude that although these orchid species were visited by different groups of visitors, few could be considered as legitimate pollinators, explaining why these tropical orchids present low values of fruit set.

**Abstract:**

A single plant might be visited by many flower visitors but not all might act as pollinators. Legitimate pollinators might also differ considerably in their efficiency, limiting pollination success. Unsuitable climatic conditions such as rain also affect pollinator activity. However, in the evergreen rainforest there is no prolonged dry season and flowering occurs usually under rain. Here, we explore the dependence on pollinators and the efficiency of flower visitors for the fruiting success of 10 Andean rainforest orchids. All species were self-compatible but strictly pollinator-dependent. Overall, we found low levels of fruit set in control flowers while experimental geitonogamous and cross-pollinations increased fruit set, revealing extensive pollination limitation in all populations. Seed viability dropped considerably after self and geitonogamous pollinations suggesting the possibility of early-acting inbreeding depression. Even though we monitored flower visitors on an extensive survey, few visitors were seen in these species and even fewer acted as legitimate pollinators. Thus, even though orchid pollination might be extremely diversified, these results show that few visitors are pollinating these species, explaining the low levels of fruit set recorded in the area studied.

## 1. Introduction

Many flower visitors are usually engaged in flower reproduction as they pick up food since while doing so, they deposit or remove pollen [1,2]. Plant–pollinator interactions might not necessarily be mutualist since some visitors can act as flower cheaters (e.g., robbers, thieves) while others might not necessarily be true pollinators or efficient enough for causing fruit set [3,4]. For instance, common flower visitors might not contribute significantly to pollination if they carry small amounts of pollen [5,6,7]. Visitors can also be efficient in pollen removal but inefficient in pollen deposition, thereby affecting pollination [8]. Thus, the concept of pollinator is theoretically easy but in practical terms might be complex because in many cases accurate determination of the effectiveness of flowers visitors as true pollinators is difficult or unfeasible [9].

Orchids constitute a good group to study the effectiveness of flower visitors to plant reproduction since a broad spectrum of visitors have been recorded, and with different rates of pollinium removal and/or deposition: hummingbirds [10], small and medium-size orchid and carpenter bees [11], nocturnal and diurnal butterflies [12,13], craneflies [14], flies [14], moths [15], and ants [16]. Visits to flowers are also constrained by the fact that more than one-third of orchid species have non-rewarding flowers, deceiving pollinators that quickly leave deceptive flowers after a few probes [17,18,19,20,21,22]. This leads to low values of fruit set especially when they have no mechanism of reproductive assurance [23,24,25,26]. Confounding flower visitors with true pollinators might lead to negative consequences. For instance, conservation efforts could be misled by suggestions that pollination networks are strong [27,28,29,30,31], or that flower visitors are acting as connectors between species [32]. Interpretations of pollination networks as either specialized or generalized can also be problematic when only visitation patterns are recorded [33].

An additional factor that constrains the activity of pollinators is the existence of unsuitable climatic conditions for their activity, such as rain [34]. In the tropical mountain rainforest, rain affects the foraging activity of pollinators, as well as the functionality and longevity of flowers, producing low reproductive-fitted individuals with fewer fruits or less viable seeds [35,36]. Pollination is, therefore, achieved by a wide range of pollinators that are more abundant during periods of low rainfall than during the wet season [37]. But in the evergreen tropical mountain rainforest there is no pronounced dry season and flowering usually occurs under rain [38,39] where unspecialized pollination mechanisms often lead to interspecific gene flow [25].

Here, we determined the ability of 10 tropical orchid species to assure fruit set through autonomous pollinations and their dependence on flower visitors. We focused our study on *Cattleya maxima* Lindl., *Epidendrum calanthum* Rchb.f. and Warsz., *Epidendrum cochlidium* Lindl., *Lycaste ciliata* (Ruiz and Pav.) Lindl. ex Rchb. f., *Masdevallia revoluta* Königer and J. Portilla, *Maxillaria lepidota* Lindl., *Oncidium excavatum* Lindley, *Phragmipedium besseae* Dodson and Kuhn, *Prosthechea fragrans* (Sw.) W.E. Higgins, and *Stelis tridentata* Lindl, which occur in small-size populations (<50 individual plants) fragmented within the south Ecuadorian evergreen rainforest. This aseasonal tropical forest has the highest known diversity of orchids in the world (more than one-tenth of all described orchids: ~4000 species [40]. Nevertheless, Ecuador has maintained the highest deforestation rates of South America during the last 20 years [41] threatening many wild populations, including orchids. Habitat changes due to agriculture expansion, wood extraction, commercial and touristic logging, mining and road construction are also important constraints for wild populations [42,43]. In situ conservation actions towards the preservation of wild orchid populations are necessary but unfeasible because we still lack information about crucial traits such as their breeding systems or the activity of pollinators. Thus, our general aim in this study was to gather basic information about these species that could be used in future conservation actions aiming to maintain wild orchid populations. Specifically, we asked: (1) Are these orchids pollinator-dependent to set fruit and seeds? (2) Do populations show evidence of pollen limitation? and (3) What is the efficiency of flower visitors as pollinators?

## 2. Materials and Methods

### 2.1. Study Species and Sites

**Figure 1:**
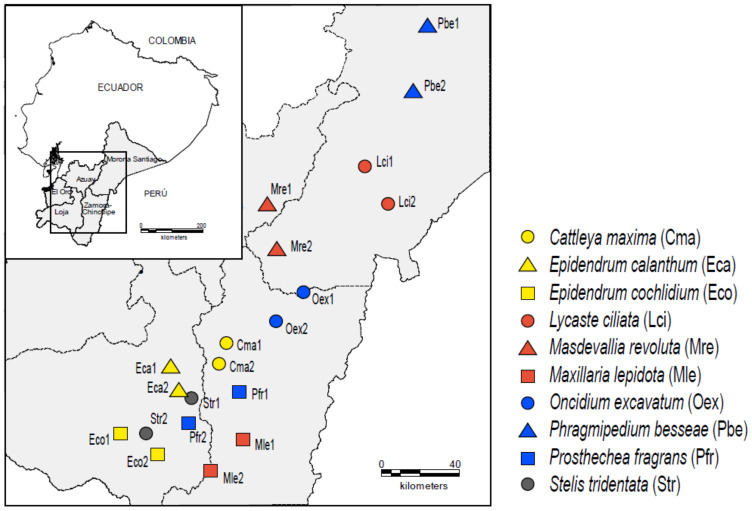
Geographic location of the 20 populations studied from the following species: *Cattleya maxima* (Cma), *Epidendrum calanthum* (Eca), *Epidendrum cochlidium* (Eco), *Lycaste ciliata* (Lci), *Masdevallia revoluta* (Mre), *Maxillaria lepidota* (Mle), *Oncidium excavatum* (Oex), *Phragmipedium besseae* (Pbe), *Prosthechea fragrans* (Pfr) and *Stelis tridentata* (Str). Two populations per species were included in this study (respectively indicated by 1 and 2).

Experiments were performed during the flowering season (October–March) of 2011–2012, 2013–2014 and 2016–2017, across 20 populations (2 populations per species; Figure 1; Table S1), which are being monitored to test the decline of pollinators. Specific geographic coordinates of orchids populations have been omitted to prevent illegal collections. All species are epiphytic in our study area except *Epidendrum calanthum*, *Epidendrum cochlidium*, *Oncidium excavatum* and *Phragmipedium besseae* that are terrestrial. Flower colours vary from light green in *Prosthechea fragrans*, green-yellow in *Lycaste ciliata* and *Stelis tridentata*, yellow in *Maxillaria lepidota*, *Oncidium excavatum*, pink in *Cattleya maxima*, *Epidendrum calanthum* and red in *Epidendrum cochlidium*, *Masdevallia revoluta*, and *Phragmipedium besseae*. Populations occur far from nearest cities in apparently well-conserved habitats. Mining and road construction are important constraints for conservation of wild orchid populations [42,43]. Illegal collection of orchids is also a concern, which is being evaluated. Fieldwork was based on an extensive walking to reach populations, coupled with night camping during several days, which allowed us to accumulate several hours of observations in a Neotropical rainforest. Our previous observations detected no nectar inside the flowers, nor floral oils or resins [39,43].

### 2.2. Breeding System

We conducted several hand-pollination treatments to investigate the breeding system of these orchids, and the presence of pollinator dependency in the populations studied. During the three flowering seasons mentioned above, inflorescences were bagged individually before the opening of flowers to prevent natural pollinations. Afterwards, each flower received one of the following treatments: (1) control, natural pollination: flowers left untouched; (2) natural self-pollination: flowers were bagged without any experimental cross; (3) experimental testing for self-pollination: flowers were hand-pollinated with the pollinium of the same flower; (4) experimental testing for geitonogamous-pollination: flowers were pollinated using the pollinium of a flower from the same individual plant; and (5) experimental testing for cross-pollination: flowers were pollinated with the pollinium collected from another individual plant. We used 20 flowers per treatment from 20 different plants. Each flower was marked, bagged after anthesis using 0.7 mm mesh nylon bags, and monitored for fruit set for 4 months. To test the possibility of late acting incompatibility, developed fruits were collected, and seeds were subsequently placed in a 1% solution of tryphenyl tetrazolium chloride and stored for 24 h at 30 ℃ to evaluate seed viability [39]. Per fruit, 250 seeds were observed under an optical microscope (100× magnification) and the percentage of viable seeds quantified.

For each species, we compared the effects of pollination treatments with a generalized linear mixed model (GLM), with pollination treatment and populations as fixed factors and year as a random effect factor, followed by post-hoc comparisons using the Scheffé’s test. Repeated measures were used since the same individuals were pollinated across years. Fruit set and seed viability were log- and square-root transformed, respectively, to meet the assumptions of normality and homoscedasticity of residuals. Additionally, a two-way ANOVA was also used to test if pollination treatments differed between years, population, and their interaction. In all cases, model assumptions, such as normality and homoscedasticity of residuals were met. For each species, pollen limitation (PL) was based on fruit set (FS) and calculated per year, as PL = 1 − (FS of control flowers/FS of cross-pollinated flowers) [44]. All statistical analyses in this study were performed with [45].

### 2.3. Flower Visitors

To compile the list of flower visitors, we monitored flowers of all orchid species in an extensive survey totaling 2016 h in the flowering season of 2011–2012 and 2376 h in the flowering season of 2016–2017, despite the recurrent rainfall and high humidity of the rainforest, which were constant during this study. Details concerning the density of individuals, observation dates, and hours per species, in each population and year are outlined in Table S1. In each population, a 2 m × 2 m plot was establish having a similar density of individuals (in the ground in case of terrestrial orchids or across trees for epiphytic orchids) within their area of occupancy. Flower visits were recorded from 09.00 to 17.00 h (Ecuador local time) using the same plots throughout the experiment, with all flowers within plots as focal units. Data were gathered in 30 min observation sessions with a total of 16 sessions each day. During all our observation days, there was never a rainless session. Observations were made using a pair of Pentax binoculars (Papilio II 6.5 × 21, Ricoh Imaging Corporation, Ltd., Tokyo, Japan) or at close range (<2 m) through rope access to the canopy, trying not to disturb bird visitors. In case insects were seen, we approached the flowers trying not to disturb the visitor. To provide the most comprehensive list of flower visitors taxa, we also carefully checked flowers, every two hours, to make sure that there were not any small insects inside, such as crane flies or micro-moths (there were none). Since flower visitors were already very low (see results), no collections were done during this study. Identifications were performed based on ongoing entomological surveys of the first author, such as the phorid fly, which has been previously identified in these populations. To clarify the efficiency of flower visitors as pollinators, we monitored the flowers every time we saw a visitor to check for pollinium deposition or removal. To exclude the possibility of nocturnal pollination, additional observations were made on ten inflorescences randomly chosen, in all populations, during the 2011–2012 and 2016–2017 flowering season, in the same days used for diurnal observations. The inflorescences were bagged diurnally from 07:00 a.m. to 19:00 p.m. and kept open nocturnally. All flowers were then checked twice (07:00 a.m. and 19:00 p.m.) to observe if any pollinium has been deposited or removed, and then followed to check the absence of fruit set, which indeed occurred in all of them. Since visits to the studied orchids were extremely infrequent (see results) we only checked for differences between populations or years using a one-way ANOVA.

## 3. Results

### 3.1. Breeding System

Fruit set of naturally pollinated flowers was generally low in all orchid species since less than half of the tested flowers set fruit (Table 1). Fruit set from natural pollinations varied significantly between species, from a minimum value of 0.15 in *Stelis tridentata* to 0.42 in *Epidendrum cochlidium* (F_1,8_ = 51.25, *p* < 0.001). Untouched bagged flowers set no fruit in any of the studied species. Experimentally self-pollinated flowers had a higher fruit set than naturally pollinated flowers in *Epidendrum calanthum* (0.37 vs. 0.33) but lower in *Epidendrum cochlidium* (0.35 vs. 0.42), *Masdevallia revoluta* (0.20 vs. 0.29), *Maxillaria lepidota* (0.17 vs. 0.25) and *Oncidium excavatum* (0.21 vs. 0.34). For the remaining species, we found no differences between naturally and experimentally self-pollinated flowers (Table 1). In all species, experimental testing for geitonogamous pollination resulted in higher fruit set than the one obtained by naturally pollinated flowers, except for *Oncidium excavatum* (F_1,12_ = 99.11; *p* < 0.001; Table 1). Nevertheless, the higher values of fruit set were always recorded after experimental cross-pollinations (F_4,24_ = 61.21; *p* < 0.001; Table 1). Fruit set from experimental cross-pollinations was significantly higher than that from naturally pollinated flowers (Table 1). Consequently, pollen limitation of fruit set was very high, varying from 0.52 in *Epidendrum cochlidium* to 0.79 in *Stelis tridentata* (Table 1). For each species, no significant differences between pollination treatments were found between years, populations, or their interaction (Table S2).

**Figure 2:**
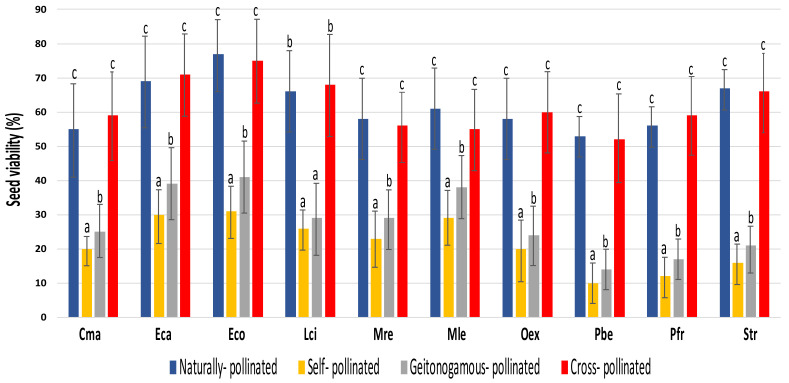
Mean seed viability (%) for the pollination treatments applied to the ten studied orchids, across three flowering seasons (2011–2012; 2013–2014; 2016–2017) and two populations per species. Mean ± SD. Superscripts denote comparisons between treatments within species using Scheffé’s test. Treatments with the same letter do not differ significantly (*p* > 0.05). Species names: *Cattleya maxima* (Cma), *Epidendrum calanthum* (Eca), *Epidendrum cochlidium* (Eco), *Lycaste ciliata* (Lci), *Masdevallia revoluta* (Mre), *Maxillaria lepidota* (Mle), *Oncidium excavatum* (Oex), *Phragmipedium besseae* (Pbe), *Prosthechea fragrans* (Pfr) and *Stelis tridentata* (Str).

In all species, seeds from experimentally self-pollinated flowers had the lowest values of seed viability (Figure 2). Seed viability increased significantly in fruits from experimentally geitonogamous pollinations (but see *Lycaste ciliata* as the exception: Table 2). However, the highest values of seed viability were recorded on seeds from naturally pollinated and experimental cross-pollinated flowers (F_4,16_ = 33.11; *p* < 0.001). No significant differences in seed viability were found between these two treatments (Figure 2). No differences in fruit set or seed viability were found between years (F_1,8_ = 2.44; *p* = 0.891; F_1,8_ = 1.03; *p* = 0.341) or between populations (F_1,4_ = 2.32; *p* = 0.872; F_1,4_ = 1.01; *p* = 0.903).

### 3.2. Flower Visitors

We only identified a total of 14 visitor taxa across all orchid species (Table 2). Hummingbirds were only recorded on the red-colored flowers of *Epidendrum cochlidium*, *Masdevallia revoluta* and *Phragmipedium besseae*. *Euglossa* bees were recorded on the green-yellow *Lycaste ciliata* and *Stelis tridentata*, the red *Masdevallia revoluta*, the green *Phragmipedium besseae*, and the yellow *Maxillaria lepidota* and *Oncidium excavatum* while other bees (*Bombus* sp. and *Xylocopa* sp.) searched the pink *Cattleya maxima* (Table 2). Butterflies were recorded on the pink *Epidendrum calanthum* (*Phoebis neocypris* and *Heliconius* sp.), the red *Epidendrum cochlidium* (*Lieinix nemesis*) and the yellow *Maxillaria lepidota* and *Oncidium excavatum* (respectively, *Heliconius* sp. and *Lieinix nemesis*). Finally, flies were seen on the green-yellow *Lycaste ciliata* (*Megaselia* sp.), the green *Prosthechea fragans* (*Scaptia* sp.) and the yellow *Oncidium excavatum* (*Scaptia* sp.).

Only one or two visitor taxa were seen in each orchid species, exceptionally three in *Maxillaria lepidota*, *Oncidium excavatum* and *Phragmipedium besseae* (Table 2). Despite our extensive field observations (2016 h in 2011–2012 and 2376 in 2015–2016 flowering season; Table S1), there was an average absence of flower visitors in 87% of our observations. In the populations studied here, species showed no evidence of nocturnal pollinations since the pollinium of diurnally bagged flowers was inside the flower, during the inspections made the next morning.

Visit duration was always very short (maximum of 19 s: Table 2). Hummingbirds usually visited flowers more rapidly than insect visitors (F_2,14_ = 55.31; *p* < 0.001) while no significant differences were found between the different groups of insect visitors (F_3,31_ = 1.11; *p* = 0.795). The frequency of visits was also very low even across flowering seasons (average: 0.11 visits/hour).

Few insects were seen visiting flowers within the same inflorescence (Table 2) suggesting a low chance for promoting geitonogamous pollinations. We also did not see any visitor facilitating self-pollinations in the monitored populations. No significant differences were found in visit duration or in the frequency of visits between populations (respectively F_2,11_ = 2.78; *p* = 0.992, F_2,34_ = 1.31; *p* = 0.905) and between years (respectively F_1,23_ = 3.17; *p* = 0.927, F_1,18_ = 2.21; *p* = 0.966).

**Figure 3:**
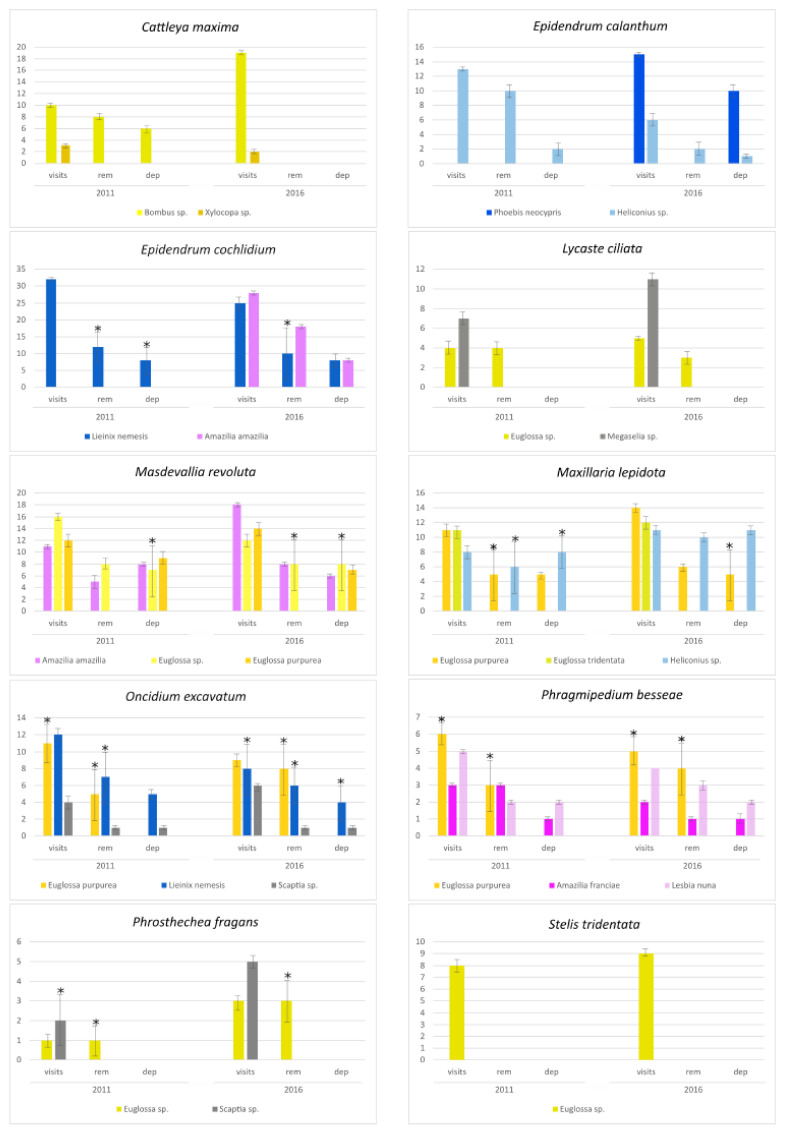
Behavior of the different flower visitors in the ten orchids studied, across two flowering seasons. Bars indicate the number of visits performed (visits), the number of pollinium removal (rem) or pollinium deposition (dep) averaged between the two studied populations, per orchid species. Error bars indicate SD between populations. Significant differences between populations were evaluated by a *t*-test and are highlighted by an asterisk. For an easier visualization of visitor types, yellow bars indicate bees, blue bars indicate butterflies, red bars indicate hummingbirds and grey bars indicate flies.

From the list of visitors recorded, few were confirmed as pollinator since some visitors did not deposit or removed any pollinium (Figure 3). For instance, the *Euglossa* bee landed on the small flowers of *Stelis tridentata* but could not reach any pollinium given the large size of this bee. This bee was also seen removing but not depositing the pollinium in several other orchid species studied (*Lycaste ciliata*, *Oncidium excavatum*, *Phragmipedium besseae* and *Prosthechea fragans*). The fly *Megaselia* sp. could not also be considered an efficient pollinator since it landed on *Lycaste ciliata* but it did not touched any pollinium (Figure 3). In fact, independent of the type of visitor, the numbers of pollinia removed or deposited were always lower than the number of visits performed, considering the two flowering seasons (Figure 3). Despite the presence of significant variation in the visitor’s behavior between populations, the results suggest that their contribution to orchid reproduction is lower than expected.

## 4. Discussion

Fruit set from naturally pollinated flowers was very low in all orchid species studied (~28%: Table 1). Although quantifications of fruiting success of orchids growing in the tropical forest are quite limited (but see [25,40,46,47]), our results support the general idea that fruit set is overall low in tropical orchids, especially if they are deceptive [23,24,26,46,47]. All orchid species studied here were self-compatible but strictly pollinator dependent as no fruits were recorded in untouched bagged flowers. Fruit set values from natural pollinations contrasted significantly with the higher values obtained through experimental pollinations (Table 1) highlighting the importance of pollinators to the fruiting success of these species. Indeed, several studies suggest that the low levels of fruit set observed in deceptive orchids are a consequence of pollinator limitation [48]. Even though deceptive orchids have developed mechanisms to promote fruiting success under conditions of pollinator limitation [49], many species are pollinator dependent [50], and when flower visits are scarce, it results in low levels of fruit set [24]. However, contrary to other plants, the low fruiting success found in most deceptive species might not necessarily affect the overall survival potential, since they can compensate the low numbers of fruit set by producing thousands of seeds per fruit [23,51]. One possible constraint found here is the fact that viability was low in seeds coming from experimental self-pollinations and from geitonogamous pollinations (Figure 2). This suggests the possibility of inbreeding depression in these orchids, at least in early life-stages. However, because inbreeding depression in early life-history traits is sometimes uncorrelated with later stages [52,53] we should interpret these results with caution. We saw very few visitors on other flowers within the same individual plant and none promoting self-pollinations, which suggests that most of the fruit set quantified in naturally pollinated flowers would come from cross-pollinations. If so, this would limit the prevalence of inbreeding depression in wild populations, a hypothesis that needs to be further investigated.

Despite intense field observations, only 14 diurnal visitor taxa were recorded across the 10 studied orchid species with no apparent association with flower colour (Table 2). From those, neither the *Euglossa* bee nor the *Megaselia* fly acted as an effective pollinator in *Stelis tridentata* and *Lycaste ciliata*, respectively, since they did not touched the pollinium (Figure 3). The *Euglossa* bee was also seen removing but not depositing the pollinium in several orchid species. Hummingbirds, which might also be efficient pollinators in neotropical areas, promoting long-distance dispersal of pollen [54], were also inefficient pollinators in the orchids studied here. In fact, despite the type of flower visitor, the numbers of pollinia removed or deposited were always lower than what might be expected considering the number of visits. In addition, no signs of nocturnal pollinations were detected in the populations studied although nocturnal pollination has been reported in several groups of orchids [55,56,57]. Thus, the overall scarce number of visitors, and the low removal and deposition of pollinia explains the low levels of fruit set that we have recorded in naturally pollinated flowers. Although we have not seen any small insects inside the flowers, future studies are being undertaken to evaluate their presence and their efficiency as pollinators. New studies involving traps are also being undertaken to better characterize the entomological community occurring in these sites. Nevertheless, even if other flower visitors are present in the populations studied here, their contribution to reproduction seems to be small due to the low values of natural fruit set observed.

To receive visits from pollinators, orchids usually take advantage of innate and behavioral biases of naïve pollinators, which quickly leave the flowering patch as they learn that the flower has no reward [21]. This explains the very short duration of the visits recorded in this study, and the fact that some visitors were not engaged in pollination. Besides the influence of deception, other general factors might also contribute to scarce visits and low efficiency of flower visitors as legitimate pollinators. For instance, flower production is often non-synchronized with pollinator emergence decreasing the effectiveness of fruit production [58]. The low pollination rate found here might also be due to the depauperation of pollinator communities since there is an overall loss and a continuous increase in the fragmentation of tropical rainforests, which contributes to reduce pollinator activity, pollen deposition and outcrossing levels in plants [59,60]. Indeed, pollinator visits tend to be reduced in small tropical forest remnants, which may have deleterious consequences for plant communities [61]. However, despite the importance of pollinators, there is a critical absence of robust species-specific estimates of pollinating groups in tropical areas, and as such we cannot generalized our results to other flowering species. For instance, general trends as for instance, historical population sizes or population dispersal rates, for which there is also an overall lack of information in orchids, cannot be ruled out. Finally, how many pollinia are being lost or wrongly delivered due to the low efficiency of flower visitors as pollinators are questions that remain to be solved. When congeneric species occur together, this unspecialized mechanism of pollination might explain why hybridization is not uncommon in certain groups of tropical orchids [24,39]. In terms of conservation, there are no current national investments for promoting wild pollinator populations, even for those useful for crop pollination, or even for starting monitoring studies such as the one reported here. The lack of standardized monitoring data on pollinators limits our understanding of species occupancy, and pollination deficit, for which conservation efforts should be developed.

## Figures and Tables

**Table 1:** Mean fruit set for the pollination treatments applied to the ten studied orchids, across three flowering seasons (2011–2012; 2013–2014; 2016–2017) and two populations per species. Crosses from natural self-pollinations are not shown as flowers did not set fruit. Mean ± SD. PL indicates pollen limitation of fruit set. Superscripts denote comparisons between treatments within species using Scheffé’s test. Treatments with the same letter do not differ significantly (*p* > 0.05).

Orchids	Naturally- Pollinated	Self- Pollinated	Geitonogamous- Pollinated	Cross- Pollinated	PL
*Cattleya maxima*	0.40 ± 0.12 ^a^	0.42 ± 0.22 ^a^	0.48 ± 0.18 ^b^	0.88 ± 0.24 ^c^	0.55
*Epidendrum calanthum*	0.33 ± 0.15 ^a^	0.37 ± 0.18 ^b^	0.45 ± 0.18 ^c^	0.87 ± 0.23 ^d^	0.62
*Epidendrum cochlidium*	0.42 ± 0.11 ^b^	0.35 ± 0.13 ^a^	0.59 ± 0.11 ^c^	0.89 ± 0.20 ^c^	0.52
*Lycaste ciliata*	0.22 ± 0.15 ^a^	0.20 ± 0.16 ^a^	0.38 ± 0.12 ^b^	0.69 ± 0.25 ^c^	0.68
*Masdevallia revoluta*	0.29 ± 0.17 ^b^	0.20 ± 0.19 ^a^	0.58 ± 0.11 ^c^	0.81 ± 0.22 ^d^	0.64
*Maxillaria lepidota*	0.25 ± 0.19 ^b^	0.17 ± 0.15 ^a^	0.47 ± 0.18 ^c^	0.83 ± 0.20 ^c^	0.69
*Oncidium excavatum*	0.34 ± 0.16 ^b^	0.21 ± 0.18 ^a^	0.31 ± 0.17 ^b^	0.79 ± 0.22 ^c^	0.56
*Phragmipedium besseae*	0.20 ± 0.09 ^a^	0.19 ± 0.10 ^a^	0.25 ± 0.12 ^b^	0.68 ± 0.31 ^c^	0.71
*Prosthechea fragrans*	0.18 ± 0.09 ^a^	0.19 ± 0.11 ^a^	0.32 ± 0.13 ^b^	0.61 ± 0.34 ^c^	0.70
*Stelis tridentata*	0.15 ± 0.10 ^a^	0.14 ± 0.12 ^a^	0.58 ± 0.15 ^b^	0.73 ± 0.56 ^c^	0.79

**Table 2:** Summary of flower visitors in the studied orchids during two flowering seasons, 2011–2012 and 2016–2017 (numbers between brackets); - indicates that the pollinator was not seen in that season. Visit duration indicates minimum and maximum length of visits in seconds (s) in 2011–2012 and 2016–2017 (numbers between brackets). % of visits to other flowers on the same individuals suggest a possible role of visitors in geitonogamous pollination (- indicates the absence of visits).

Orchids	Habit	Flower Colour	Pollinator Group	Flower Visitors	Visit Duration (s)	% of Visits to Other Flowers
*Cattleya maxima*	Epiphytic	Pink	Bee	*Bombus* sp.	8–12 (7–12)	40%
			Bee	*Xylocopa* sp.	5–9 (5–10)	-
*Epidendrum calanthum*	Terrestrial	Pink	Butterfly	*Phoebis neocypris*	- (11–15)	-
			Butterfly	*Heliconius* sp.	12–19 (13–18)	20%
*Epidendrum cochlidium*	Terrestrial	Red	Butterfly	*Lieinix nemesis*	14–18 (11–16)	20%
			Hummingbird	*Amazilia amazilia*	- (5–8)	-
*Lycaste ciliata*	Epiphytic	Green-yellow	Bee	*Euglossa* sp.	9–12 (9–11)	-
			Fly	*Megaselia* sp.	10–14 (8–13)	-
*Masdevallia revoluta*	Epiphytic	Red	Hummingbird	*Amazilia amazilia*	5–8 (3–6)	-
			Bee	*Euglossa* sp.	7–10 (8–12)	50%
			Bee	*Euglossa purpurea*	8–11(9–14)	30%
*Maxillaria lepidota*	Epiphytic	Yellow	Bee	*Euglossa purpurea*	8–13 (9–15)	40%
			Bee	*Euglossa tridentata*	9–15 (11–17)	30%
			Butterfly	*Heliconius* sp.	11–15 (14–18)	-
*Oncidium excavatum*	Terrestrial	Yellow	Bee	*Euglossa purpurea*	9–12 (9–13)	-
			Butterfly	*Lienix nemesis*	14–18 (-)	-
			Fly	*Scaptia* sp.	9–13 (8–14)	2%
*Phragmipedium besseae*	Terrestrial	Red	Hummingbird	*Amazilia franciae*	5–7 (8–10)	-
			Hummingbird	*Lesbia nuna*	6–9 (7–10)	-
			Bee	*Euglossa purpurea*	7–11 (8–13)	-
*Prosthechea fragrans*	Epiphytic	Light green	Fly	*Scaptia* sp.	5–9 (6–10)	2%
			Bee	*Euglossa* sp.	11 (12–15)	-
*Stelis tridentata*	Epiphytic	Green	Bee	*Euglossa* sp.	10–13 (11–14)	-

## Data Availability

Data is contained within the article or supplementary material.

## Supplementary Materials

The following are available online at https://www.mdpi.com/article/10.3390/insects12100856/s1. Table S1: Details of the study performed in the ten deceptive orchids to quantify the level of reproductive success. Dates of observation of insects and the corresponding total number of hours in two flowering seasons (2011–2012 and 2016–2017) are indicated. Table S2: Results of generalized linear mixed model concerning pollination treatments across populations, years, and the interaction populations x years. No significant value has been obtained.
